# The Effect of Extreme Response and Non-extreme Response Styles on Testing Measurement Invariance

**DOI:** 10.3389/fpsyg.2017.00726

**Published:** 2017-05-23

**Authors:** Min Liu, Allen G. Harbaugh, Jeffrey R. Harring, Gregory R. Hancock

**Affiliations:** ^1^Educational Psychology, University of HawaiiHonolulu, HI, USA; ^2^Education Leadership and Policy Studies Cluster, School of Education, Boston UniversityBoston, MA, USA; ^3^Human Development and Quantitative Methodology, University of MarylandCollege Park, MD, USA

**Keywords:** extreme response style, non-extreme response style, measurement invariance, cross-group comparison, survey research

## Abstract

Extreme and non-extreme response styles (RSs) are prevalent in survey research using Likert-type scales. Their effects on measurement invariance (MI) in the context of confirmatory factor analysis are systematically investigated here via a Monte Carlo simulation study. Using the parameter estimates obtained from analyzing a 2007 Trends in International Mathematics and Science Study data set, a population model was constructed. Original and contaminated data with one of two RSs were generated and analyzed via multi-group confirmatory factor analysis with different constraints of MI. The results indicated that the detrimental effects of response style on MI have been underestimated. More specifically, these two RSs had a substantially negative impact on both model fit and parameter recovery, suggesting that the lack of MI between groups may have been caused by the RSs, not the measured factors of focal interest. Practical implications are provided to help practitioners to detect RSs and determine whether RSs are a serious threat to MI.

## Introduction

In the social and behavioral sciences, research instruments using Likert-type scales are often applied to study and compare individuals in different cultures or other well-defined groups. However, if there is significant variation among the groups with regard to how given response scales are interpreted, any findings of similarity or difference among the groups with regard to the intended content of the survey instrument may not be valid. That is to say, comparisons across groups may result in invalid results and possibly incorrect conclusions. This necessitates an examination of the degree to which the scale is measuring the same construct or trait across these groups, that is, whether a given measurement scale could be interpreted in the same way for the respondents from different groups. Exploring or testing a research hypothesis about group differences is only meaningful once measurement invariance (MI) based on a given instrument has been well-established. However, many empirical studies using either confirmatory factor analysis (CFA) or item response theory (IRT) to assess MI have overlooked response styles (Van Vaerenbergh and Thomas, 2013). Response styles (RSs) are a source of measurement error (or bias) that occurs when respondents tend to provide answers not based on the substantive meaning of the questionnaire items but on content-irrelevant factors. Two common and relatively easily identifiable RSs are (1) the extreme response style (ERS) in which a participant is inclined to mark only the extreme ends of the scale, and (2) the non-extreme response style (NERS) in which a participant is inclined to systematically avoid selecting the extreme ends of the scale. Most MI studies that we have reviewed neither mentioned RSs nor examined their potential effects on the statistical models (e.g., Marsh et al., 2012; review study by Vandenberg and Lance, 2000). Although MI and RSs are not new measurement problems to researchers, only a few studies investigated both of them in the same setting (Cheung and Rensvold, 2000; Moors, 2004; Kankaraš and Moors, 2009, 2011). Moreover, none of these empirical studies have ever systematically investigated whether RSs have a detrimental effect on the MI in cross-group studies. The current study examines the hypotheses that ERS and NERS, two RSs that are frequently seen in practice, have a significant effect on MI and that their effects on the levels of MI may vary depending on different percentages of RSs present in the data. This paper is organized as follows. First, the literature review includes a brief introduction to MI as defined in a multi-group CFA model, a bit more detail on RSs, and then a survey of the few studies that have tried to address both measurement problems simultaneously. Second, a simulation study is presented in which we examine whether different percentages of ERS and NERS underlying data have detrimental effects on MI. Finally, the last section presents implications for practitioners and future directions for research based on the findings.

### Measurement invariance

Researchers often use survey data with Likert-type scales to measure and compare subjects' attitudes, perceptions, evaluations, and beliefs across nations, ethnicities, or other segregable groups (e.g., Khine, 2008). All group comparison studies are based on a critical assumption that the instrument operates in exactly the same manner for all of the possible different groups defined by the variable of interest (e.g., gender, ethnicity, or other geographic variables). This assumption suggests that the items of the survey are measuring the same constructs for each group, the items are not showing differential item functioning (DIF) from one group to another (i.e., the possibility of a group × content interaction), and the response scales are being used and interpreted by members of each group in the same manner. More generally, it is assumed that MI of the instrument holds across these groups. Early research examining MI was based on studying factor invariance over several decades (e.g., Thurstone, 1947; Meredith, 1964). More recently, a number of researchers have developed formal procedures for testing MI across groups using multi-group CFA (Jöreskog, 1971; Horn and McArdle, 1992; Meredith, 1993; Millsap and Olivera-Aguilar, 2012). Several widely-available software packages, LISREL (Jöreskog and Sörbom, 2015), EQS (Bentler, 2008), AMOS (Arbuckle, 2012), and Mplus (Muthén and Muthén, 2015), facilitate application of the CFA models in examining MI efficiently and widely in various disciplines. A brief search of the key word “measurement invariance” and “multigroup confirmatory factor analysis” in Google Scholar brought up more than 100 and 400 publications in 2014 and 2015, respectively, and a rough review of these studies indicated that checking MI through multigroup CFA in cross-group studies had been used as a routine preliminary step prior to conducting group mean comparisons.

In the psychometrics literature, Meredith (1993) formulated four levels of hierarchy for cross-group MI ranging from low to high. These levels are (1) configural invariance (CI)—same “shape” for factor model specification across groups; (2) weak invariance (WI)—same factor loadings across groups, also known as metric invariance (Horn and McArdle, 1992); (3) strong invariance (SI)—same factor loadings and intercepts across groups, also known as scalar invariance; and (4) strict invariance (STRI)—same factor loadings, intercepts, and residual variances across groups. With the 2nd level of WI, differences across groups in relationships among measured variables are ascribed to differences across groups in relationships among latent variables. With the 3rd level of SI, group differences in co-variances among measured variables and in means of measured variables are ascribed to group differences in co-variances and means on latent variables. Under strong factorial invariance, group differences in those variances of measured variables could be ascribed to group differences in variances of latent variables as well as to group differences in error variances. With the 4th level of STRI, group differences in variances of measured variables are ascribed only to group differences in variances of latent variables, since error variances are invariant across groups. Although the 2nd and 3rd levels are commonly accepted as sufficient evidence for MI (e.g., Little, 1997; Vandenberg and Lance, 2000), some researchers suggest that the 4th level is a necessary condition for MI (Meredith, 1993; Wu et al., 2007). As our focal concern is not to address this debate, we sequentially investigate MI following this hierarchical structure through all four levels. It is worth noting that levels 3 and 4 may be defined differently for categorical indicators. According to Millsap and Yun-Tein (2004), factor loadings and thresholds are constrained to be equal across groups in level 3 and the additional constraints of equal residual variances are necessary for level 4.

### Response styles

Numerous studies have shown that data collected through survey instruments using Likert-type scales may be subject to different forms of response bias. One source of response bias is the use of different RSs among different subpopulations. According to Baumgartner and Steenkamp (2001), RSs are also referred to as response sets or response biases. Specifically, response bias is defined by Paulhus (1991) as a systematic tendency to respond to questions on some basis that is independent of the content of the question. Messick (1962) suggested that Likert-type scales demonstrated this type of systematic measurement error, stating that response bias was “confounded with legitimate replies to item content” (p. 41) resulting in threats to the validity of scales. Furthermore, the response bias caused by consistent personal styles or traits may be stable and reliable components of responses (Messick, 1962). Thus, RSs may account for another source of variation common to most survey data, a source that could be included in the statistical models.

In cross-cultural research in fields like marketing (Baumgartner and Steenkamp, 2001) and education (Lam and Klockars, 1982; Bond and Hwang, 1986), it is well-noted that respondents from different cultural backgrounds may show systematically different response patterns that are content-irrelevant. For example, American respondents may tend to select the extreme endpoints of non-frequency Likert-type scales more often than Japanese or Chinese peers (Chen et al., 1995). As Baumgartner and Steenkamp (2001) summarized, several types of RSs may occur frequently in cross-cultural or other group comparison studies. A few of these include: (1) ERS, the tendency to select the extreme endpoints of a scale; (2) NERS, the tendency to avoid selecting the extreme endpoints of a scale resulting in only selecting the very middle or the middle-most values of a scale; (3) acquiescence response style (ARS), the tendency to agree with all items regardless of content; and (4) disacquiescence response style (DRS), the tendency to disagree with all items. The pervasiveness of these types of RSs has been evidenced by a growing body of research (e.g., Costa and McCrae, 1992; Rost et al., 1997; Buckley, 2009). In this paper, ERS and NERS are the primary focus because they are the most frequently studied RSs in the social sciences (e.g., Paulhus, 1991; Barnette, 1999; Baumgartner and Steenkamp, 2001; Johnson, 2003) and both appear to be present in a large-scale data set used in this study (see method section for more details).

As Cavusgil and Das (1997) pointed out, the manifestation of RSs in one's data can jeopardize the statistical and external validity of the research as well as affect comparability across samples. While a number of previous studies have examined Likert-type data and the associated response bias, these studies have focused more on practical implications for survey instrument builders such as the optimal number of response categories or the effect of response bias on the reliability and validity of measures (e.g., Barnette, 1999; Preston and Colman, 2000; Liu et al., 2010). Except for Liu and colleagues' (2010) work modeling outliers, none of these previous studies attempted to systematically investigate the effects of the RSs from a statistical modeling perspective. In other words, we still do not know whether (or to what extent) the RSs have a detrimental effect in statistical modeling such as MI in the context of multi-group CFA, nor how serious it could be under different bias conditions. When statistical models derived from sound conceptual theory do not fit the data, researchers commonly explore the modeling space to find an acceptable model. It may be that—despite apparent model-data misfit—the model or latent construct is fine, once accounting for the group-specific differences; the real problem may be an issue of analyzing data “contaminated” by RSs.

Although applied researchers are aware of the possibility that both construct non-equivalence and RSs may distort the comparison of attitudes across groups, only a few studies investigated these two measurement issues simultaneously. Cheung and Rensvold (2000) suggested using multi-group CFA to test for the presence of ERS and ARS and to determine whether group comparison studies on a basis of latent factors are meaningful. As Morren et al. (2011) summarized, this method is useful to determine whether MI is invalidated by a RS. However, it can neither measure the extent of the impact of the RSs, nor correct for the presence of this RS, nor rule out the possibility that non-invariant intercepts and thresholds may still be caused by content-related factors (e.g., DIF). Moors (2003, 2004) proposed a latent class factor analysis (LCFA) approach to detect and correct for ERS, which was modeled as a latent factor in addition to the factors of interest. Although some researchers recommend LCFA because it requires fewer assumptions about the factor capturing the variation brought about by RS (e.g., Morren et al., 2011), it is still unclear whether this method is effective in detecting and correcting ERS. Moreover, none of these researchers investigated the effects of RSs from a statistical modeling perspective.

Little to no reporting on the effects of RSs on MI is found in educational research. This may be attributed to a lack of familiarity with various RSs that have been identified under different names, uncertainty about how to deal with RSs appropriately due to insufficient research in comparing the efficiencies of the proposed methods, or more importantly, the pervasive belief that the RSs do not have substantively deleterious effects on the validity of the statistical analyses used by researchers (Baumgartner and Steenkamp, 2001). Therefore, a series of systematic simulation studies is necessary to address the concern that statistical conclusions from measurement models may be spurious due to RSs and eventually to find out which method is most appropriate to deal with various RSs present in a data set. The current study is an attempt to initiate the former part of this systematic process: this study aims to investigate whether two RSs, ERS and NERS, have substantial effects on construct comparability among different groups. If they do, it would be critical to determine what percentages of contaminated data have detrimental effects on MI. Moreover, Lombardi and Pastore's (2012) examined the performance of structural equation model (SEM) based fit indices in analyzing data with faked observations caused by dishonest responses from participants in answering survey questionnaire. They found that incremental fit indices, such as the Comparative Fit Index (CFI) and the Tucker Lewis Index [TLI, also named as the Non-Normed Fit Index (NNFI)], are more sensitive to the faked observations when participants are not responding honestly. Therefore, another research goal of the current study is to investigate which SEM-based model fit indices are more sensitive to the inclusion of contaminated cases. In sum, the findings of the current study will be informative to practitioners who are interested in applying MI when their data may be suspected to be influenced by RSs.

## Methods

Using parameter estimates from an authentic data source, data sets were simulated to represent that which might reasonably be expected from an authentic population. Using a model-based algorithm, contamination via ERS and NERS was introduced into the simulated data sets. The extent to which the congeneric factor analysis model measuring a single latent factor (self-concept in this case) exhibits MI between two groups was investigated with Mplus 7.4 (Muthén and Muthén, 2015). The weighted least squares estimator (WLSMS) and THETA parameterization were used to estimate all the models. DIFFEST was requested to obtain model comparisons between models with different levels of MI.

### Generating raw and contaminated data

**Figure 1:**
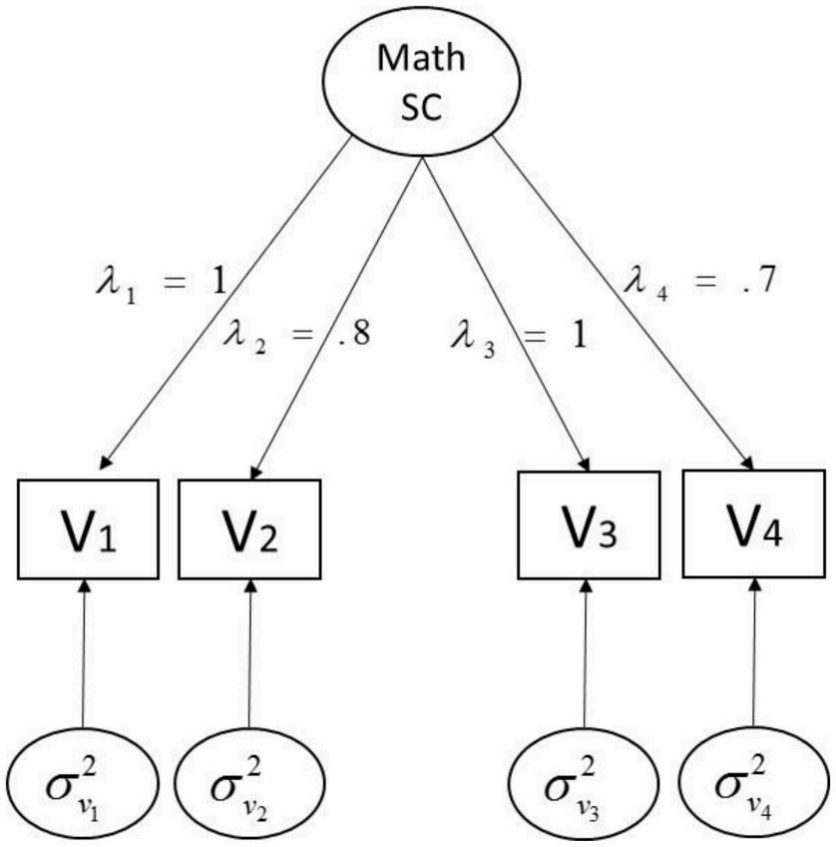
**Population model for simulation**. λs represent the factor loadings [1, 0.8, 1, 0.7] for V1–V4; σ^2^s denote the residual variances for V1–V4, and thresholds τs are [0, 2, 3] for V1, [0, 1, 1.5] for V2, and [0, 1, 2] for V3 and V4, respectively.

Our simulation design is based on parameter estimates from a two-group (i.e., U.S. and Hong Kong) factor analysis model for four selected Trends in International Mathematics and Science Study (TIMSS) measures of students' math cognitive self-concept (Mullis et al., 2011). The details of the population model and associated parameters are depicted in Figure 1. As our primary concern is to investigate how ERS and NERS contamination affect four levels of hierarchy for cross-group MI, STRI holds in the true population model; factor loadings, thresholds, and residual variances are equal across two groups, of which the values come from U.S. TIMSS data. To vary the population condition for the two groups, the factor mean and factor variance were respectively fixed to 0 and 1.6 for one group (i.e., U.S. students) and 0.9 and 1 for the other (i.e., Hong Kong students) according to estimates based on the TIMSS data. As WLSMS is known to require large sample size (Boomsma and Hoogland, 2001), *n* = 3,000 observations for each group is fixed to minimize sampling variability and thus obtain reliable results. One thousand data sets were generated for each manipulated condition.

As indicated above, the current study focuses on ERS and NERS because they are more often seen in reality and clearly prevalent in TIMSS data (see Table 1). We found 20% of the U.S. students chose the end points only while more than 10 percent of Hong Kong (HK) students always selected the middle points for all four of the survey items. The proportion of ERS and NERS are the primary manipulated factor to address our research question. More specifically, if a U.S. respondent has a tendency of ERS, a response pattern of {1,2,4,3} would be expected to convert to {1,1,4,4}, whereas the same response pattern for a HK respondent with a tendency of NERS would convert to {2,2,3,3}. Seven different combined proportions of RS contamination, 5 vs. 5%, 10 vs. 10%, 20 vs. 20%, 10 (HK) vs. 20% (USA), 20 (HK) vs. 30 (USA), 30 vs. 30%, and 40 vs. 40%, were used to transform the raw data into contaminated data with one of the two RSs for one group, i.e., ERS was applied to USA group and NERS was applied to Hong Kong group. This attempts to model the assumption that students in U.S. and HK exhibit ERS and NERS, respectively, in survey items, which has been studied and confirmed by many researchers (e.g., Chen et al., 1995).

**Table 1:** **Percentages of responses to four TIMSS survey items using four-point Likert scales from USA and Hong Kong**.

**TIMSS survey items**	**USA**				**Hong Kong**			
	**Agree a lot**	**Agree a little**	**Disagree a little**	**Disagree a lot**	**Agree a lot**	**Agree a little**	**Disagree a little**	**Disagree a lot**
V1. I usually do well in math	45.2	42.6	7	3.4	21.9	57.6	16.9	2.9
V2. Math is harder for me	13.5	19.7	21.6	42.6	9	23.1	39.2	27.5
V3. I am not good at math	9.1	13.7	16.9	57.8	20	27.5	26.5	25.9
V4. I learn things quickly in math	36.3	38.5	14.4	7.9	22.3	42.9	27.9	6.8

### Conducting measurement invariance studies

Following Millsap and Yun-Tein's (2004) guidelines, we proceeded to examine the four levels of MI using the generated data. A few general constraints were imposed for the purpose of scale identification. First, the factor loading of the reference indicator was fixed to 1 whereas other factors loadings were freely estimated. Second, one threshold for each indicator was equally constrained across groups and one additional threshold for the reference indicator was equally constrained across groups. That is to say, two thresholds were constrained for the reference indicator, and one threshold was constrained for all other indicators. Third, the residual variances of the U.S. group were fixed to 1 and those of the HK group were freely estimated. The last general constraint was that the factor mean of the U.S. group was fixed to 0 across all the models. Next, as stated in last section, different sets of parameters were constrained across the models to assess the four levels of MI.

### Determining the effects of ERS and NERS

We applied the CFA model with four levels of MI to analyze eight sets of simulated data (authentic data and data contaminated with seven different percentages of ERS/NERS cases) and obtained a total of 32 sets of estimated results, based on which we could determine the extent of the detrimental effects of different proportions of ERS and NERS to the MI results. As our focus is to address these research concerns from a statistical modeling perspective, we are particularly interested in summarizing aspects of the results to evaluate RS effects from the following two perspectives:

1. Model-data fit. To evaluate the effect on the model-data fit introduced by different RSs, several global model fit indices will be compared: Chi-square test, Chi-square difference test, relative Chi-square (also called normal Chi-square, the ratio of Chi-square test to degree freedom), CFI, TLI, Root Mean Square Error of Approximation (RMSEA), and Weighted Root-Mean-square Residual (WRMR).
2. Parameter recovery. Since additional systematic measurement error is introduced by RSs, we expect that parameter estimates from contaminated data will be more biased as compared with those from less contaminated ones. In our CFA models, the focus will be on the factor loading parameters. The accuracy of each estimated parameter was quantified using bias. Bias is an average difference between true and estimated parameters over all replications:
  $$\begin{array}{l} bias=\frac{1}{n}\overset{n}{\underset{j=1}{\sum }}\left({\hat{\theta }}_{j}-\theta_{j}\right) \end{array}$$

where θ_*j*_ is the true value of a parameter, and ${\hat{\theta }}_{j}$ is the estimated value of the parameter for the *j*-th replication over a total *n* replications. To gauge the variability of parameter estimates, 90% confidence intervals were also included.

## Results

All the models were estimated appropriately except a few replications in the least constrained model imposing only CI. We hypothesized the non-convergent replications may be caused by insufficient sample size or poor starting values because the model with CI requires estimation of more parameters and no starting values were used in our simulation study. As our focal interest is to examine the effects of ERS and NERS on the statistical modeling results, we summarized estimated results in tables in terms of their effects on model fit to data and parameters estimates separately.

### RS effects on model fit indices

Tables 2A,B presents the average model fit results over 1,000 replications for the original and contaminated data (totally eight conditions) in the four levels of MI: CI, WI, SI, and STRI. M0–0 (or M0) represents the model being fitted to the uncontaminated data for both groups and M10–20 represents the models being fitted to one group of data with 10 percentages of NERS and to the other group of data with 20 percentages of ERS. Other models could be interpreted in the same way. Inspecting Tables 2A,B, we found the estimated results based on original (i.e., uncontaminated) data achieved almost perfect model fit to data, as evidenced by the non-significant Chi-square test value, the small ratio of Chi-square test to degree of freedom, perfect CFI, TLI, RMSEA, and WRMR in all M0 results in all four levels of MI models. Moreover, the Chi-square difference testing results also indicates that the restriction of equal factor loadings, equal thresholds, and equal residuals variances do not degrade the model fit to data. Actually the ratio of Chi-square test to degree of freedom decreased, indicating better fit. This result is consistent with the true population model in which STRI holds.

**Table 2A:** **Model fit results from models with four types of measurement invariance across eight different percentages of contaminated data (Conditions 1–4)**.

**Model fit estimates**	**M0–0**	**M5–5**	**M10–10**	**M20–20**
**Average**	**CI**			
χ^2^ (*df* = 4)	5.81 (1.94, 10.75)	5.47 (1.78, 10.11)	4.47 (1.16, 8.62)	5.40 (1.40, 10.59)
*p*-value	0.34 (0.03, 0.75)	0.38 (0.04, 0.78)	0.46 (0.07, 0.88)	0.40 (0.03, 0.84)
% (*p* < 0.05)	4%	12%	8%	13%
χ^2^/*df*	1.45 (0.49, 2.69)	1.37 (0.44, 2.53)	1.11 (0.29, 2.13)	1.33 (0.35, 2.65)
% (χ^2^/*df* < 5)	99.35%	99.18%	99.70%	98.80%
CFI	1.00 (1.00, 1.00)	1.00 (1.00, 1.00)	1.00 (1.00, 1.00)	1.00 (1.00, 1.00)
TLI	1.00 (1.00, 1.00)	1.00 (1.00, 1.00)	1.00 (1.00, 1.00)	1.00 (1.00, 1.00)
RMSEA	0.01 (0, 0.02)	0.01 (0, 0.02)	0.01 (0, 0.02)	0.01 (0, 0.02)
WRMR	0.39 (0.24, 0.56)	0.37 (0.22, 0.53)	0.33 (0.18, 0.49)	0.35 (0.19, 0.53)
**Average**	**WI**			
χ^2^ (*df* = 7)	5.64 (2.43, 9.86)	6.18 (2.45, 10.69)	7.4 (3.22, 12.26)	9.25 (3.96, 16.29)
*p*-value	0.61 (0.20, 0.93)	0.57 (0.15, 0.93)	0.46 (0.09, 0.86)	0.36 (0.02, 0.78)
% (*p* < 0.05)	0%	3%	6%	16%
Δχ^2^(*df* = 3)	0.75 (0.06, 2.18)	1.54 (0.14, 3.57)	3.08 (0.43, 6.28)	3.99 (0.89, 7.84)
*p*-value	0.86 (0.54, 0.99)	0.72 (0.31, 0.99)	0.49 (0.10, 0.93)	0.39 (0.05, 0.83)
% (*p* < 0.05)	0%	1%	4%	10%
χ^2^/*df*	0.81 (0.35, 1.41)	0.88 (0.35, 1.53)	1.06 (0.46, 1.75)	1.32 (0.57, 2.33)
% (χ^2^/*df* < 5)	100%	100%	100%	99.90%
CFI	1.00 (1.00, 1.00)	1.00 (1.00, 1.00)	1.00 (1.00, 1.00)	1.00 (1.00, 1.00)
TLI	1.00 (1.00, 1.00)	1.00 (1.00, 1.00)	1.00 (1.00, 1.00)	1.00 (1.00, 1.00)
RMSEA	0.00 (0.00, 0.01)	0.00 (0.00, 0.01)	0.01 (0.00, 0.02)	0.01 (0.00, 0.02)
WRMR	0.39 (0.28, 0.58)	0.43 (0.28, 0.60)	0.48 (0.32, 0.64)	0.51 (0.35, 0.71)
**Average**	**SI**			
χ^2^(*df* = 14)	9.66 (5.68, 14.21)	11.44 (6.71, 16.65)	16.53 (11.01, 22.81)	27.69 (18.13, 37.71)
*p*-value	0.76 (0.43, 0.97)	0.65 (0.28, 0.95)	0.35 (0.06, 0.69)	0.07 (0.00, 0.20)
% (*p* < 0.05)	0%	1%	7%	**67%**
Δχ^2^(*df* = 7)	4.10 (2.13, 6.51)	5.45 (2.65,8.54)	9.11 (5.49,13.11)	18.14 (10.67,25.88)
*p*-value	0.76 (0.48, 0.95)	0.62 (0.29, 0.92)	0.30 (0.07, 0.60)	**0.05 (0.00, 0.15)**
% (*p* < 0.05)	0%	1%	7%	**74%**
χ^2^/*df*	0.69 (0.41, 1.01)	0.82 (0.48, 1.19)	1.18 (0.79, 1.63)	1.98 (1.29, 2.69)
% (χ^2^/*df* < 5)	99.90%	99.90%	99.90%	99.90%
CFI	1.00 (1.00, 1.00)	1.00 (1.00, 1.00)	1.00 (1.00, 1.00)	1.00 (1.00, 1.00)
TLI	1.00 (1.00, 1.00)	1.00 (1.00, 1.00)	1.00 (1.00, 1.00)	1.00 (1.00, 1.00)
RMSEA	0.00 (0.00, 0.00)	0.00 (0.00, 0.01)	0.01 (0, 0.02)	0.02 (0.01, 0.03)
WRMR	0.57 (0.42, 0.72)	0.61 (0.46, 0.77)	0.75 (0.61, 0.91)	**0.93 (0.75**, **1.10)**
**Average**	**STRI**			
χ^2^ (*df* = 18)	11.72 (7.54, 16.69)	21.4 (14.41, 28.72)	77.35 (63.06, 93.07)	142.32 (121.45, 165.57)
*p*-value	0.82 (0.54, 0.98)	0.33 (0.05, 0.70)	**0 (0, 0)**	**0 (0, 0)**
% (*p* < 0.05)	0%	10%	**100%**	**100%**
Δχ^2^(*df* = 4)	1.98 (0.57, 3.85)	9.67 (4.64, 15.57)	58.92 (45.77, 72.11)	108.36 (89.87, 127.77)
*p*-value	0.74 (0.43, 0.97)	0.12 (0.00, 0.33)	**0 (0, 0)**	**0 (0, 0)**
% (*p* < 0.05)	0%	47%	**100%**	**100%**
χ^2^/*df*	0.65 (0.42, 0.93)	1.19 (0.80, 1.60)	4.30 (3.50, 5.17)	**7.91 (6.75**, **9.20)**
% (χ^2^/*df* < 5)	99.90%	99.90%	73.30%	**0.1%**
CFI	1.00 (1.00, 1.00)	1.00 (1.00, 1.00)	1.00 (1.00, 1.00)	1.00 (1.00, 1.00)
TLI	1.00 (1.00, 1.00)	1.00 (1.00, 1.00)	1.00 (1.00, 1.00)	1.00 (1.00, 1.00)
RMSEA	0 (0, 0)	0.01 (0, 0.01)	0.03 (0.03, 0.04)	0.05 (0.04, 0.05)
WRMR	0.64 (0.50, 0.79)	0.87 (0.71, 1.03)	**1.72 (1.56**, **1.89)**	**2.22 (2.05**, **2.39)**

*Averages are listed with 10th and 90th percentile ranges indicated in parentheses; M0–0, M5–5, M10–10, M20–20, M10–20, M20–30, M30–30, and M40–40 indicates models estimated with certain percent contaminated data for two groups; Indices suggesting unsatisfactory model fit indicated in bold*.

**Table 2B:** **Model fit results from models with four types of measurement invariance across eight different percentages of contaminated data (Conditions 5–8)**.

**Model fit**	**M10–20**	**M20–30**	**M30–30**	**M40–40**
**Average**	**CI**			
χ^2^ (*df* = 4)	4.60 (1.23, 9.27)	5.26 (1.36,10.25)	6.08 (1.49, 12.23)	6.98 (1.42,14.70)
*p*-value	0.46 (0, 0.87)	0.41 (0.04, 0.85)	0.37 (0.02, 0.83)	0.34 (0.01, 0.84)
% (*p* < 0.05)	9.10%	12.40%	18%	24%
χ^2^/*df*	1.15 (0.31, 2.32)	1.32 (0.34, 2.56)	1.52 (0.37, 3.06)	1.74 (0.35, 3.67)
% (χ^2^/*df* < 5)	99.60%	98.80%	98.20%	96.40%
CFI	1 (1, 1)	1 (1, 1)	1 (1, 1)	1 (1, 1)
TLI	1 (1, 1)	1 (1, 1)	1 (1, 1)	1 (1, 1)
RMSEA	0.01 (0, 0.02)	0.01 (0, 0.02)	0.01 (0, 0.03)	0.01 (0, 0.03)
WRMR	0.32 (0.18, 0.49)	0.34 (0.18, 0.51)	0.36 (0.19, 0.56)	0.38 (0.18, 0.60)
**Average**	**WI**			
χ^2^ (*df* = 7)	7.39 (3.06, 13.16)	9.59 (4.03, 16.48)	12 (5.16, 20.82)	14.73 (6.53, 24.28)
*p*-value	0.48 (0.07, 0.88)	0.35 (0.02, 0.78)	0.24 (0.00, 0.64)	0.16 (0.00, 0.48)
% (*p* < 0.05)	6.70%	18.80%	31%	48%
Δχ^2^ (*df* = 3)	2.91 (0.62, 5.73)	4.29 (0.90, 8.22)	5.77 (1.43, 10.84)	7.52 (2.14, 14.10)
*p*-value	0.50 (0.13, 0.89)	0.37 (0.04, 0.82)	0.26 (0.01, 0.70)	0.18 (0.00, 0.54)
% (*p* < 0.05)	4.10%	11.90%	24%	40%
χ^2^/*df*	1.06 (0.44, 1.88)	1.37 (0.58, 2.35)	1.71 (0.74, 2.97)	2.10 (0.93, 3.47)
% (χ^2^/*df* < 5)	99.90%	99.90%	99.30%	97.10%
CFI	1.00 (1.00, 1.00)	1.00 (1.00, 1.00)	1.00 (1.00, 1.00)	1.00 (1.00, 1.00)
TLI	1.00 (1.00, 1.00)	1.00 (1.00, 1.00)	1.00 (1.00, 1.00)	1.00 (1.00, 1.00)
RMSEA	0.01 (0, 0.02)	0.01 (0, 0.02)	0.01 (0.01, 0.03)	0.02 (0.01, 0.03)
WRMR	0.46 (0.30, 0.64)	0.51 (0.34, 0.69)	0.57 (0.38, 0.78)	0.61 (0.42, 0.81)
**Average**	**SI**			
χ^2^ (*df* = 14)	24.86 (16.33, 34.00)	35.87 (24.48, 47.56)	42.66 (29.75, 65.20)	58.35 (43.18, 74.74)
*p*-value	0.11 (0.00, 0.29)	**0.02 (0, 0.04)**	**0 (0, 0.01)**	**0 (0, 0.00)**
% (*p* < 0.05)	**54.40%**	**92.80%**	**99%**	**100%**
Δχ^2^ (*df* = 7)	17.12 (10.05, 24.38)	26.11 (16.74, 35.55)	30.49 (20.49, 40.96)	44.48 (32.09, 57.68)
*p*-value	0.06 (0.00, 0.19)	**0.01 (0, 0.02)**	**0 (0, 0.00)**	**0 (0, 0.00)**
% (*p* < 0.05)	**68%**	**96.30%**	**100%**	**100%**
χ^2^/*df*	1.78 (1.17, 2.43)	2.56 (1.75, 3.40)	3.05 (2.13, 4.01)	4.17 (3.08, 5.34)
% (χ^2^/*df* < 5)	99.90%	99.90%	98.10%	64.80%
CFI	1.00 (1.00, 1.00)	1.00 (1.00, 1.00)	1.00 (1.00, 1.00)	1.00 (1.00, 1.00)
TLI	1.00 (1.00, 1.00)	1.00 (1.00, 1.00)	1.00 (1.00, 1.00)	1.00 (1.00, 1.00)
RMSEA	0.02 (0.01, 0.02)	0.02 (0.02, 0.03)	0.03 (0.02, 0.03)	**0.08 (0.07**, **0.09)**
WRMR	0.88 (0.71, 1.05)	**1.01 (0.84, 1.18)**	**1.10 (0.92, 1.28)**	**1.22 (1.05**, **1.39)**
**Average**	**STRI**			
χ^2^ (*df* = 18)	56.93 (43.76, 71)	142.39 (120.59, 166.35)	274.36 (242.99, 306.85)	419.28 (380.01, 458.87)
*p*-value	**0 (0**, **0)**	**0 (0**, **0)**	**0 (0, 0)**	**0 (0, 0)**
% (*p* < 0.05)	**99.80%**	**100%**	**100%**	**100%**
Δχ^2^ (*df* = 4)	30.98 (21.14, 42.22)	98.93 (80.73, 117.49)	211.28 (185.80, 237.63)	312.01 (282.08, 342.41)
*p*-value	**0 (0**, **0)**	**0 (0**, **0)**	**0 (0, 0)**	**0 (0, 0)**
% (*p* < 0.05)	**99.90%**	**100%**	**100%**	**100%**
χ^2^/*df*	3.16 (2.43, 3.94)	**7.91 (6.70**, **9.24)**	**15.24 (13.50**, **17.05)**	**23.29 (21.11**, **25.49)**
% (χ^2^/*df* < 5)	99.70%	**0%**	**0%**	**0%**
CFI	1.00 (1.00, 1.00)	0.99 (0.99, 1.00)	0.99 (0.98, 0.99)	0.99 (.98, 0.99)
TLI	1.00 (1.00, 1.00)	0.99 (0.99, 1.00)	0.99 (0.99, 0.99)	0.99 (0.99, 0.99)
RMSEA	0.03 (0.02, 0.03)	0.05 (0.04, 0.05)	**0.07 (0.07, 0.08)**	**0.09 (0.08**, **0.09)**
WRMR	**1.39 (1.22**, **1.56)**	**2.13 (1.97**, **2.31)**	**2.97 (2.80,3.15)**	**3.53 (3.36**, **3.70)**

*Averages are listed with 10th and 90th percentile ranges indicated in parentheses; M0–0, M5–5, M10–10, M20–20, M10–20, M20–30, M30–30, and M40–40 indicates models estimated with certain percent contaminated data for two groups; Indices suggesting unsatisfactory model fit indicated in bold*.

In contrast, the model constraints may slightly or substantially decrease model fit using the data contaminated with RSs, as evidenced by the other seven conditions from M5 through M40 across all four levels of MI. In general, CI of this factor analysis model holds for the contaminated data although some model fit indices show increasingly worse fit to data as the percentage of contamination goes up. But the results show the models fit data adequately well.

After equal factor loadings were imposed on the model, as the WI part in Table 2 shows, most model-data fit indices are worse than their corresponding CI models except CFI and TLI. When the proportion of contamination reaches 40%, the probabilities of obtaining significant Chi-square test and Chi-square difference test results increase to 48 and 40% respectively, resulting in higher likelihoods of rejecting the WI model. However, the model fit results are still generally acceptable.

When both equal factor loadings and equal thresholds are imposed on the model (i.e., SI), as shown in the SI part of Table 2, 5 percent of cases with RS does not seem to have a serious effect on model fit as they achieve a satisfactory fit to data; in the M10 models, 10% of contaminated cases increase the chance of achieving a significant Chi-square test and Chi-square difference test above 0.05. Additionally, data sets with 20% contamination result in Chi-square tests and Chi-square difference tests that are significant more than half of what is expected by chance alone, and moreover, the resulting average WRMR of 0.93 exceeds the desired cut score. In the two conditions of unequal percentages of contamination, 10% (NERS) vs. 20% (ERS) and 20% (NERS) vs. 30% (ERS), Chi-square test and Chi-square difference tests tend to be significant more than half of the time. In the latter condition of 20 vs. 30%, WRMR has an average value of 1.01 with 90% confidence interval of 0.84 and 1.18. These results do not support the claim that the models fit the data well. When the percentage of contamination reaches 30%, the Chi-square tests and Chi-square difference tests are always significant, indicating the SI model does not fit the data and the SI model is statistically significantly worse than the WI model; the average value of WRMR increases to 1.10 and almost all of WRMR exceed 0.9. When the percentage reaches 40%, except for the CFI and TLI, all the other model fit indices, including RMSEA, show unacceptable fit to the data. The Chi-square to df ratio suggests that the models do not fit the data about 35% of the time.

The tolerance of STRI models to the contaminated data is even lower. Five percent contamination results in the chance of obtaining statistically significant Chi-square tests and Chi-square difference tests; 10 percent contamination leads to significant Chi-square and Chi-square difference tests and an unsatisfactory average WRMR of 1.72 with 90 percent of confidence interval above 1.56. When the percentage contamination increases to 20 (NERS) and 30 (ERS), most model fit indices indicate unacceptable fit to data except CFI, TLI, and RMSEA. When the percentage of contaminated cases is 30 or above, RMSEA also suggests unsatisfactory model fit results.

### Sensitiveness of model fit indices to RSs

According to Tables 2A,B, clearly, the SEM model fit indices that were examined in the current study exhibit different degrees of sensitiveness to the inclusion of contaminated cases. CFI and TLI are the least sensitive measures as they always indicate a perfect fit to data regardless of how many percentage of NERS and ERS cases were included. RMSEA only indicates misfit for SI and STRI models when the percentage of cases goes up to 40 percent. As compared with CFI, TLI, and RMSEA, Chi-square test, Chi-square difference test, and relative Chi-square test are more sensitive to the inclusion of two types of RSs to the data. Using the suggested ratio of 5 (Jackson et al., 1993, p. 755), relative Chi-square test may alert misfit for STRI models when the percentages of contamination are 20% or above. Both Chi-square and Chi-square difference tests are more likely (above 50%) to indicate unfit/less fit for SI models than WI models when the percentage of contamination is 20% or above. Only 10% of contaminated cases would result in STRI models being rejected by the Chi-square and Chi-square difference tests. Among all the fit indices, WRMR may be the most sensitive one to the contaminated cases as it shows misfit for SI models when the percentages of NERS and ERS cases are 20% or above and for STRI models when the percentages reach 10%.

### RS effects on parameter estimates

The results of parameter estimates from models with four levels of MI were summarized in Table 3 through **Table 6**, respectively. All the tables have the same structure. The first two columns list the parameter to be estimated and the true population values for those parameters. As indicated above, M0–0 to M40–40 represent models estimated from data with percentages of contamination varying from 0 to 40% for two groups. Under each model, the bias of parameter estimates and 90% confidence intervals are presented in three columns to evaluate the quality of the parameter estimates. For the non-contaminated data, the estimated parameter results are presented in the M0 section, we found estimation bias in the least residual constrained CI models, in which thresholds are underestimated and residual variances are overestimated for one group, most likely due to CI being the most complicated model, and thus requiring more parameters to be estimated. The bias in other more constrained models, WI, SI, and STRI models, is negligible.

**Table 3A:** **Summary of parameter estimates for models with configural invariance across eight different percentages of contaminated data (Conditions 1–4)**.

**Configure invariance**		**M0–0**			**M5–5**			**M10–10**			**M20–20**		
**Parameters**	**True value**	**Bias**	**90% CI of estimates**		**Bias**	**90% CI of estimates**		**Bias**	**90% CI of estimates**		**Bias**	**90% CI of estimates**	
τ11	0.00	0.00	−0.07	0.06	0.07	0.00	0.13	0.07	0.05	0.08	0.31	0.23	0.39
τ12	2.00	0.01	1.89	2.13	0.11	1.99	2.24	0.27	2.26	2.27	0.47	2.31	2.64
τ13_US	3.00	0.02	2.84	3.18	0.04	2.86	3.22	0.26	3.26	3.27	0.25	3.05	3.46
τ21	0.00	0.00	−0.05	0.05	0.05	0.00	0.11	0.05	0.04	0.06	0.25	0.18	0.32
τ22_US	1.00	0.00	0.94	1.08	0.06	0.99	1.13	0.00	1.00	1.00	0.27	1.19	1.35
τ23_US	1.50	0.01	1.43	1.59	0.06	1.47	1.64	0.00	1.49	1.50	0.25	1.66	1.85
τ31	0.00	0.00	−0.06	0.06	0.06	0.00	0.13	0.13	0.13	0.14	0.29	0.21	0.36
τ32_US	1.00	0.00	0.92	1.09	0.07	0.99	1.15	0.06	1.06	1.06	0.31	1.22	1.41
τ33_US	2.00	0.01	1.90	2.12	0.06	1.95	2.18	−0.08	1.92	1.92	0.27	2.15	2.40
τ41	0.00	0.00	−0.05	0.04	0.05	0.00	0.10	0.05	0.04	0.06	0.24	0.18	0.31
τ42_US	1.00	0.00	0.94	1.07	0.06	1.00	1.13	0.03	1.03	1.03	0.29	1.21	1.37
τ43_US	2.00	0.00	1.92	2.09	0.04	1.95	2.13	−0.01	1.99	1.99	0.21	2.11	2.31
λ2_US	0.80	0.00	0.72	0.90	−0.02	0.70	0.87	−0.13	0.67	0.67	−0.05	0.68	0.82
λ3_US	1.00	0.00	0.89	1.13	−0.03	0.87	1.08	−0.22	0.78	0.78	−0.10	0.81	0.99
λ4_US	0.70	0.00	0.63	0.78	0.00	0.64	0.78	−0.08	0.62	0.62	0.01	0.65	0.78
σ^2^(F)_US	1.60	0.02	1.37	1.90	0.44	1.73	2.37	0.86	2.45	2.46	2.18	3.23	4.42
τ13_HK	3.00	0.02	2.82	3.22	0.14	2.94	3.36	0.38	3.29	3.48	0.59	3.34	3.87
τ22_HK	1.00	−0.15	0.36	0.96	0.39	0.41	3.88	1.00	0.51	6.54	0.76	1.15	2.81
τ23_HK	1.50	−0.22	0.55	1.44	0.58	0.62	5.81	1.57	0.77	9.97	1.15	1.69	4.32
τ32_HK	1.00	−0.16	0.60	1.13	0.16	0.37	3.18	4.32	0.91	17.01	0.50	1.05	2.25
τ33_HK	2.00	−0.32	1.16	2.21	0.25	0.71	6.22	8.42	1.69	33.49	0.70	1.85	4.13
τ42_HK	1.00	−0.14	0.56	1.06	0.53	0.54	5.08	1.92	0.65	9.52	1.12	1.26	3.59
τ43_HK	2.00	−0.28	1.12	2.10	0.98	1.04	10.22	3.72	1.23	18.72	1.90	2.29	6.73
λ2_HK	0.80	−0.12	0.29	0.77	0.20	0.31	2.72	0.55	0.34	4.30	0.21	0.67	1.61
λ3_HK	1.00	−0.17	0.57	1.11	0.02	0.33	2.75	3.35	0.74	14.04	0.00	0.71	1.48
λ4_HK	0.70	−0.10	0.38	0.76	0.28	0.35	3.16	1.05	0.39	5.69	0.40	0.69	1.87
σ^2^(v1)_HK	1.00	0.01	0.89	1.13	−0.01	0.87	1.11	0.09	0.99	1.19	−0.07	0.81	1.07
σ^2^(v2)_HK	1.00	0.58	0.14	0.89	6.36	0.13	12.26	6.74	0.17	32.13	0.73	0.55	4.38
σ^2^(v3)_HK	1.00	0.60	0.32	1.23	2.47	0.09	7.89	60.86	0.47	227.27	0.08	0.41	2.52
σ^2^(v4)_HK	1.00	0.20	0.32	1.11	6.80	0.21	24.34	20.67	0.28	74.26	1.76	0.73	7.67
μ(F)_HK	0.90	0.00	0.85	0.96	0.12	0.96	1.09	0.23	1.08	1.18	0.51	1.32	1.51
σ^2^(F)_HK	1.00	0.01	0.87	1.17	−0.02	0.82	1.14	0.06	0.95	1.17	−0.07	0.76	1.12

*The parameters not labeled with US or HK are constrained equal across the two groups*.

**Table 3B:** **Summary of parameter estimates for models with configural invariance across eight different percentages of contaminated data (Conditions 5–8)**.

**Configure invariance**		**M10–20**			**M20–30**			**M30–30**			**M40–40**		
**Parameters**	**True value**	**Bias**	**90% CI of estimates**		**bias**	**90% CI of estimates**		**Bias**	**90% CI of estimates**		**Bias**	**90% CI of estimates**	
τ11	0.00	0.31	0.23	0.39	0.55	0.45	0.64	0.55	0.45	0.64	0.88	0.75	1.01
τ12	2.00	0.47	2.30	2.64	0.77	2.58	2.98	0.77	2.58	2.98	1.15	2.91	3.41
τ13_US	3.00	0.25	3.05	3.45	0.48	3.27	3.73	0.48	3.27	3.73	0.82	3.56	4.13
τ21	0.00	0.25	0.18	0.31	0.43	0.35	0.50	0.43	0.35	0.50	0.66	0.57	0.75
τ22_US	1.00	0.27	1.19	1.35	0.45	1.36	1.56	0.45	1.36	1.55	0.70	1.58	1.82
τ23_US	1.50	0.25	1.66	1.85	0.43	1.82	2.04	0.43	1.82	2.04	0.67	2.03	2.30
τ31	0.00	0.29	0.21	0.37	0.49	0.40	0.59	0.49	0.41	0.59	0.76	0.66	0.88
τ32_US	1.00	0.31	1.22	1.41	0.53	1.42	1.65	0.53	1.42	1.65	0.81	1.69	1.95
τ33_US	2.00	0.27	2.15	2.40	0.47	2.33	2.62	0.47	2.33	2.62	0.74	2.58	2.92
τ41	0.00	0.24	0.18	0.31	0.42	0.35	0.49	0.42	0.35	0.49	0.66	0.57	0.74
τ42_US	1.00	0.29	1.21	1.37	0.48	1.39	1.58	0.48	1.39	1.58	0.74	1.63	1.86
τ43_US	2.00	0.21	2.11	2.31	0.37	2.26	2.50	0.37	2.26	2.50	0.60	2.47	2.75
λ2_US	0.80	−0.05	0.68	0.82	−0.07	0.66	0.81	−0.07	0.66	0.81	−0.08	0.65	0.80
λ3_US	1.00	−0.10	0.81	0.99	−0.12	0.79	0.96	−0.12	0.79	0.96	−0.15	0.77	0.95
λ4_US	0.70	0.01	0.64	0.78	0.01	0.65	0.78	0.01	0.65	0.78	0.01	0.64	0.79
σ^2^(F)_US	1.60	2.18	3.23	4.42	3.98	4.77	6.57	3.98	4.77	6.58	6.77	7.10	9.96
τ13_HK	3.00	0.57	3.32	3.85	0.92	3.64	4.24	0.94	3.66	4.26	1.38	4.03	4.75
τ22_HK	1.00	0.56	1.05	2.30	0.74	1.38	2.19	0.87	1.46	2.43	1.11	1.78	2.53
τ23_HK	1.50	0.77	1.51	3.45	1.01	1.97	3.21	1.28	2.12	3.66	1.56	2.55	3.73
τ32_HK	1.00	0.41	0.98	2.01	0.65	1.32	2.05	0.72	1.38	2.16	1.02	1.73	2.39
τ33_HK	2.00	0.52	1.74	3.68	0.78	2.20	3.53	0.92	2.30	3.76	1.24	2.73	3.92
τ42_HK	1.00	0.81	1.12	2.82	1.01	1.52	2.69	1.22	1.62	3.08	1.38	1.95	2.96
τ43_HK	2.00	1.33	2.02	5.28	1.52	2.62	4.84	1.89	2.79	5.53	1.96	3.16	5.08
λ2_HK	0.80	0.11	0.64	1.33	0.06	0.69	1.06	0.11	0.72	1.15	0.05	0.73	1.00
λ3_HK	1.00	−0.03	0.70	1.39	−0.07	0.75	1.14	−0.06	0.77	1.17	−0.10	0.78	1.06
λ4_HK	0.70	0.26	0.61	1.45	0.21	0.71	1.21	0.29	0.75	1.36	0.20	0.75	1.11
σ^2^(v1)_HK	1.00	0.04	0.90	1.19	−0.02	0.84	1.14	−0.12	0.75	1.02	−0.18	0.70	0.95
σ^2^(v2)_HK	1.00	0.52	0.55	3.49	0.19	0.62	2.07	0.21	0.60	2.23	−0.02	0.59	1.63
σ^2^(v3)_HK	1.00	0.11	0.44	2.37	−0.09	0.49	1.60	−0.15	0.45	1.51	−0.28	0.43	1.20
σ^2^(v4)_HK	1.00	1.26	0.67	5.29	0.79	0.83	3.50	0.92	0.82	3.93	0.40	0.76	2.48
μ(F)_HK	0.90	0.45	1.26	1.44	0.78	1.56	1.80	0.84	1.62	1.87	1.26	2.00	2.34
σ^2^(F)_HK	1.00	0.04	0.86	1.26	−0.02	0.79	1.20	−0.13	0.71	1.08	−0.19	0.64	1.01

*The parameters not labeled with US or HK are constrained equal across the two groups*.

#### Factor loadings λs

*Factor loadings* λ*s* are our primary concern as they represent the relationship between the latent factor and its manifested indicators; they are comparable to slopes in regression analysis, in which factors are predictors and indicators are dependent variables. Therefore, they are the first and most important criteria in evaluating MI using CFA. Inspecting M0 sections across all the four tables, we found that—except for the slightly underestimated factor loadings of the HK group (λ_*i*_ HK) in the CI model—all the other factor loadings in the models with CI or above are appropriately estimated via the original data because all the average estimates over 1,000 replications are nearly identical to the true population values. When the data included contaminated cases, as reflected by M5 to M40 in Tables 3–6, nearly all the estimates of the factor loadings in the models are biased. In general, the absolute bias increases with the percentage of contaminated cases. It is worth noting that the bias is substantial and could not be ignored.

**Table 4A:** **Summary of parameter estimates for models with weak invariance across eight different percentages of contaminated data (Conditions 1–4)**.

**Weak invariance**		**M0–0**			**M5–5**			**M10–10**			**M20–20**		
**Parameters**	**True**	**Bias**	**90% CI of estimates**		**bias**	**90% CI of estimates**		**Bias**	**90% CI of estimates**		**Bias**	**90% CI of estimates**	
τ11	0.00	0.00	−0.07	0.06	0.07	0.00	0.13	0.08	0.05	0.10	0.33	0.24	0.41
τ12	2.00	0.00	1.89	2.13	0.11	1.99	2.24	0.26	2.26	2.27	0.46	2.30	2.63
τ13_US	3.00	0.01	2.84	3.18	0.04	2.87	3.22	0.26	3.25	3.26	0.25	3.05	3.44
τ21	0.00	0.00	−0.06	0.06	0.06	0.00	0.11	0.06	0.04	0.08	0.24	0.17	0.31
τ22_US	1.00	0.00	0.94	1.08	0.06	0.99	1.14	0.00	1.00	1.00	0.27	1.19	1.35
τ23_US	1.50	0.01	1.43	1.60	0.06	1.47	1.65	0.00	1.49	1.50	0.25	1.66	1.85
τ31	0.00	0.00	−0.06	0.07	0.07	0.01	0.13	0.11	0.09	0.13	0.30	0.22	0.37
τ32_US	1.00	0.00	0.92	1.08	0.07	0.99	1.16	0.06	1.06	1.06	0.31	1.22	1.41
τ33_US	2.00	0.00	1.89	2.11	0.06	1.95	2.17	−0.08	1.92	1.92	0.27	2.15	2.40
τ41	0.00	0.00	−0.05	0.05	0.05	0.00	0.10	0.05	0.03	0.08	0.22	0.16	0.28
τ42_US	1.00	0.00	0.93	1.06	0.06	1.00	1.13	0.03	1.03	1.03	0.29	1.21	1.37
τ43_US	2.00	0.00	1.92	2.09	0.04	1.96	2.13	−0.01	1.99	2.00	0.21	2.11	2.31
λ2	0.80	0.01	0.72	0.90	−0.01	0.70	0.87	−0.13	0.67	0.67	−0.05	0.68	0.82
λ3	1.00	0.01	0.90	1.13	−0.03	0.87	1.08	−0.22	0.78	0.78	−0.10	0.81	0.99
λ4	0.70	0.00	0.63	0.78	0.01	0.64	0.78	−0.08	0.62	0.62	0.01	0.65	0.78
σ^2^(F)_US	1.60	0.00	1.37	1.87	0.42	1.73	2.36	0.84	2.44	2.46	2.17	3.23	4.39
τ13_HK	3.00	0.01	2.82	3.21	0.14	2.94	3.35	0.37	3.28	3.47	0.57	3.33	3.83
τ22_HK	1.00	0.01	0.91	1.11	0.07	0.98	1.17	0.00	0.96	1.05	0.31	1.22	1.41
τ23_HK	1.50	0.01	1.39	1.64	0.11	1.49	1.73	0.02	1.45	1.59	0.44	1.83	2.06
τ32_HK	1.00	0.00	0.90	1.12	0.08	0.99	1.19	−0.03	0.92	1.01	0.37	1.26	1.48
τ33_HK	2.00	0.01	1.85	2.19	0.09	1.93	2.26	−0.16	1.76	1.91	0.41	2.26	2.58
τ42_HK	1.00	0.00	0.92	1.09	0.08	0.99	1.18	0.04	0.99	1.09	0.36	1.27	1.46
τ43_HK	2.00	0.01	1.87	2.15	0.11	1.96	2.26	0.00	1.90	2.10	0.46	2.31	2.61
σ^2^(v1)_HK	1.00	0.01	0.89	1.13	0.49	0.87	1.11	0.58	0.97	1.18	0.41	0.79	1.04
σ^2^(v2)_HK	1.00	0.01	0.87	1.14	0.43	0.81	1.05	0.22	0.62	0.84	0.26	0.67	0.87
σ^2^(v3)_HK	1.00	0.01	0.87	1.16	0.42	0.78	1.05	0.10	0.52	0.70	0.25	0.64	0.85
σ^2^(v4)_HK	1.00	0.01	0.88	1.16	0.47	0.86	1.11	0.29	0.68	0.91	0.37	0.77	0.99
μ(F)_HK	0.90	0.00	0.85	0.95	0.12	0.95	1.09	0.24	1.10	1.19	0.52	1.32	1.53
σ^2^(F)_HK	1.00	0.01	0.87	1.17	−0.02	0.84	1.12	0.02	0.93	1.11	−0.12	0.75	1.03

*The parameters not labeled with US or HK are constrained equal across the two groups*.

**Table 4B:** **Summary of parameter estimates for models with weak invariance across eight different percentages of contaminated data (Conditions 5–8)**.

**Weak invariance**		**M10–20**			**M20–30**			**M30–30**			**M40–40**		
**Parameters**	**True**	**Bias**	**90% CI of estimates**		**Bias**	**90% CI of estimates**		**Bias**	**90% CI of estimates**		**Bias**	**90% CI of estimates**	
τ11	0.00	0.32	0.24	0.41	0.58	0.47	0.68	0.58	0.48	0.69	0.93	0.80	1.06
τ12	2.00	0.46	2.31	2.63	0.76	2.57	2.97	0.76	2.57	2.96	1.13	2.91	3.37
τ13_US	3.00	0.25	3.05	3.44	0.48	3.27	3.72	0.47	3.26	3.72	0.80	3.54	4.08
τ21	0.00	0.24	0.18	0.31	0.42	0.34	0.49	0.41	0.33	0.49	0.64	0.55	0.73
τ22_US	1.00	0.27	1.19	1.35	0.45	1.36	1.56	0.45	1.36	1.56	0.70	1.58	1.82
τ23_US	1.50	0.25	1.66	1.85	0.43	1.82	2.04	0.43	1.82	2.04	0.67	2.04	2.30
τ31	0.00	0.29	0.22	0.37	0.51	0.42	0.59	0.51	0.42	0.60	0.79	0.68	0.90
τ32_US	1.00	0.31	1.22	1.41	0.53	1.42	1.64	0.53	1.42	1.64	0.81	1.69	1.95
τ33_US	2.00	0.27	2.15	2.40	0.47	2.33	2.62	0.47	2.33	2.61	0.74	2.58	2.91
τ41	0.00	0.23	0.17	0.29	0.39	0.32	0.46	0.39	0.32	0.46	0.61	0.53	0.70
τ42_US	1.00	0.29	1.21	1.37	0.49	1.39	1.59	0.49	1.39	1.59	0.75	1.63	1.86
τ43_US	2.00	0.21	2.11	2.31	0.38	2.26	2.50	0.38	2.26	2.50	0.61	2.48	2.75
λ2	0.80	−0.05	0.68	0.82	−0.06	0.67	0.81	−0.06	0.67	0.81	−0.07	0.66	0.80
λ3	1.00	−0.10	0.81	0.99	−0.12	0.79	0.96	−0.12	0.79	0.96	−0.14	0.78	0.95
λ4	0.70	0.01	0.65	0.78	0.02	0.65	0.79	0.02	0.65	0.79	0.02	0.65	0.79
σ^2^(F)_US	1.60	2.17	3.23	4.40	3.95	4.75	6.53	3.94	4.75	6.52	6.65	7.04	9.71
τ13_HK	3.00	0.56	3.31	3.82	0.89	3.62	4.20	0.90	3.63	4.20	1.31	3.98	4.64
τ22_HK	1.00	0.28	1.18	1.38	0.49	1.39	1.60	0.53	1.42	1.63	0.80	1.68	1.93
τ23_HK	1.50	0.35	1.72	1.96	0.63	2.00	2.26	0.73	2.10	2.36	1.07	2.41	2.73
τ32_HK	1.00	0.31	1.20	1.43	0.57	1.45	1.70	0.62	1.50	1.75	0.94	1.81	2.10
τ33_HK	2.00	0.32	2.16	2.49	0.61	2.45	2.79	0.70	2.53	2.88	1.06	2.88	3.26
τ42_HK	1.00	0.34	1.25	1.44	0.57	1.47	1.68	0.59	1.49	1.70	0.88	1.76	2.01
τ43_HK	2.00	0.43	2.28	2.58	0.70	2.54	2.86	0.73	2.57	2.89	1.06	2.89	3.24
σ^2^(v1)_HK	1.00	0.03	0.89	1.17	0.45	0.82	1.10	0.34	0.73	0.97	0.27	0.65	0.90
σ^2^(v2)_HK	1.00	−0.13	0.76	0.99	0.27	0.67	0.88	0.18	0.60	0.78	0.12	0.54	0.71
σ^2^(v3)_HK	1.00	−0.17	0.71	0.95	0.24	0.63	0.86	0.16	0.56	0.76	0.09	0.50	0.69
σ^2^(v4)_HK	1.00	0.01	0.88	1.14	0.43	0.81	1.05	0.31	0.71	0.92	0.24	0.64	0.84
μ(F)_HK	0.90	0.46	1.26	1.46	0.79	1.57	1.83	0.86	1.63	1.89	1.28	2.02	2.35
σ^2^(F)_HK	1.00	0.01	0.86	1.18	−0.07	0.78	1.09	−0.19	0.69	0.96	−0.26	0.62	0.87

*The parameters not labeled with US or HK are constrained equal across the two groups*.

**Table 5A:** **Summary of parameter estimates for models with strict invariance across eight different percentages of contaminated data (Conditions 1–4)**.

**Strict invariance**		**M0–0**			**M5–5**			**M10–10**			**M20–20**		
**Parameters**	**True**	**Bias**	**90% CI of estimates**		**Bias**	**90% CI of estimates**		**Bias**	**90% CI of estimates**		**Bias**	**90% CI of estimates**	
τ11	0.00	0.00	−0.06	0.06	0.05	−0.02	0.11	0.04	0.01	0.07	0.26	0.17	0.34
τ12	2.00	0.00	1.91	2.10	0.10	2.00	2.21	0.18	2.12	2.24	0.48	2.34	2.62
τ13	3.00	0.00	2.87	3.14	0.11	2.97	3.25	0.23	3.14	3.32	0.53	3.36	3.71
τ21	0.00	0.00	−0.05	0.05	0.03	−0.02	0.09	0.01	−0.01	0.03	0.17	0.10	0.24
τ22	1.00	0.00	0.94	1.07	0.07	1.00	1.14	0.05	1.02	1.08	0.32	1.24	1.42
τ23	1.50	0.00	1.43	1.58	0.10	1.53	1.68	0.11	1.58	1.65	0.48	1.87	2.08
τ31	0.00	0.00	−0.06	0.06	0.04	−0.02	0.10	0.06	0.04	0.08	0.22	0.14	0.30
τ32	1.00	0.00	0.93	1.08	0.08	1.00	1.16	0.10	1.07	1.14	0.39	1.28	1.50
τ33	2.00	0.00	1.91	2.11	0.11	2.02	2.22	0.14	2.08	2.19	0.53	2.40	2.67
τ41	0.00	0.00	−0.05	0.05	0.03	−0.02	0.08	0.02	−0.01	0.04	0.16	0.10	0.22
τ42	1.00	0.00	0.94	1.06	0.07	1.00	1.14	0.07	1.04	1.10	0.33	1.25	1.41
τ43	2.00	0.00	1.92	2.08	0.09	2.00	2.17	0.10	2.06	2.14	0.42	2.32	2.52
λ2	0.80	0.00	0.74	0.87	−0.01	0.73	0.86	−0.06	0.70	0.78	−0.03	0.70	0.84
λ3	1.00	0.00	0.92	1.09	−0.02	0.90	1.07	−0.07	0.87	0.98	−0.05	0.86	1.03
λ4	0.70	0.00	0.64	0.76	0.00	0.64	0.76	−0.03	0.64	0.71	0.01	0.65	0.77
σ^2^(F)_US	1.60	0.01	1.41	1.82	0.46	1.79	2.32	0.59	2.03	2.36	2.43	3.53	4.60
μ(F)_HK	0.90	0.00	0.85	0.96	0.10	0.93	1.06	0.17	1.01	1.13	0.46	1.27	1.46
σ^2^(F)_HK	1.00	0.00	0.88	1.14	0.00	0.88	1.13	0.05	0.95	1.17	−0.02	0.86	1.12

*The parameters not labeled with US or HK are constrained equal across the two groups*.

**Table 5B:** **Summary of parameter estimates for models with strong invariance across eight different percentages of contaminated data (Conditions 5–8)**.

**Strong invariance**		**M10–20**			**M20–30**			**M30–30**			**M40–40**		
**Parameters**	**True**	**Bias**	**90% CI of estimates**		**Bias**	**90% CI of estimates**		**Bias**	**90% CI of estimates**		**Bias**	**90% CI of estimates**	
τ11	0.00	0.34	0.25	0.42	0.60	0.50	0.70	0.61	0.50	0.72	0.98	0.83	1.11
τ12	2.00	0.37	2.23	2.52	0.63	2.45	2.83	0.64	2.46	2.84	0.97	2.75	3.21
τ13	3.00	0.33	3.13	3.54	0.61	3.38	3.87	0.64	3.40	3.91	0.99	3.70	4.30
τ21	0.00	0.25	0.19	0.32	0.43	0.36	0.50	0.42	0.35	0.50	0.65	0.57	0.74
τ22	1.00	0.24	1.17	1.32	0.41	1.32	1.50	0.40	1.31	1.49	0.63	1.53	1.74
τ23	1.50	0.27	1.67	1.86	0.46	1.85	2.08	0.48	1.87	2.10	0.75	2.12	2.38
τ31	0.00	0.31	0.23	0.38	0.52	0.43	0.61	0.52	0.43	0.61	0.81	0.70	0.91
τ32	1.00	0.30	1.21	1.40	0.51	1.40	1.63	0.51	1.40	1.62	0.79	1.67	1.92
τ33	2.00	0.28	2.15	2.40	0.47	2.32	2.61	0.47	2.33	2.61	0.74	2.58	2.90
τ41	0.00	0.24	0.18	0.30	0.41	0.34	0.48	0.40	0.33	0.47	0.63	0.55	0.72
τ42	1.00	0.26	1.19	1.34	0.45	1.37	1.54	0.45	1.37	1.54	0.70	1.60	1.81
τ43	2.00	0.23	2.14	2.34	0.42	2.31	2.53	0.41	2.30	2.53	0.66	2.53	2.80
λ2	0.80	−0.05	0.69	0.82	−0.07	0.66	0.80	−0.09	0.64	0.78	−0.11	0.62	0.77
λ3	1.00	−0.08	0.84	1.01	−0.12	0.79	0.96	−0.15	0.77	0.94	−0.17	0.74	0.91
λ4	0.70	0.00	0.65	0.77	0.00	0.63	0.76	−0.01	0.62	0.75	−0.02	0.62	0.76
σ^2^(F)_US	1.60	2.14	3.21	4.32	4.08	4.86	6.67	4.29	5.04	6.95	7.31	7.54	10.48
σ^2^(v1)_HK	1.00	−0.11	0.80	0.98	−0.19	0.72	0.91	−0.27	0.64	0.81	−0.37	0.55	0.72
σ^2^(v2)_HK	1.00	−0.22	0.70	0.86	−0.38	0.55	0.70	−0.49	0.45	0.57	−0.58	0.38	0.47
σ^2^(v3)_HK	1.00	−0.21	0.70	0.88	−0.37	0.56	0.72	−0.48	0.46	0.59	−0.57	0.37	0.48
σ^2^(v4)_HK	1.00	−0.17	0.76	0.90	−0.30	0.65	0.77	−0.40	0.55	0.65	−0.49	0.46	0.56
μ(F)_HK	0.90	0.41	1.22	1.41	0.73	1.51	1.76	0.79	1.56	1.83	1.19	1.93	2.27
σ^2^(F)_HK	1.00	−0.12	0.76	1.02	−0.23	0.66	0.90	−0.33	0.57	0.79	−0.42	0.49	0.68

*The parameters not labeled with US or HK are constrained equal across the two groups*.

**Table 6A:** **Summary of parameter estimates for models with strong invariance across eight different percentages of contaminated data (Conditions 1–4)**.

**Strong invariance**		**M0–0**			**M5–5**			**M10–10**			**M20–20**		
**Parameters**	**True**	**Bias**	**90% CI of estimates**		**Bias**	**90% CI of estimates**		**Bias**	**90% CI of estimates**		**Bias**	**90% CI of estimates**	
τ11	0.00	0.00	−0.06	0.06	0.07	0.00	0.13	0.09	0.06	0.11	0.34	0.25	0.43
τ12	2.00	0.00	1.90	2.11	0.09	1.97	2.20	0.20	2.16	2.24	0.38	2.24	2.54
τ13	3.00	0.01	2.85	3.16	0.07	2.91	3.24	0.24	3.18	3.29	0.36	3.16	3.59
τ21	0.00	0.00	−0.05	0.05	0.05	0.00	0.11	0.06	0.04	0.09	0.25	0.18	0.31
τ22	1.00	0.00	0.94	1.07	0.05	0.99	1.12	0.00	0.98	1.01	0.23	1.16	1.31
τ23	1.50	0.00	1.43	1.58	0.06	1.48	1.64	0.00	1.49	1.51	0.28	1.69	1.88
τ31	0.00	0.00	−0.06	0.06	0.07	0.01	0.12	0.11	0.09	0.14	0.31	0.23	0.38
τ32	1.00	0.00	0.93	1.08	0.07	1.00	1.14	0.05	1.03	1.06	0.30	1.21	1.40
τ33	2.00	0.00	1.90	2.11	0.06	1.95	2.18	−0.04	1.94	1.97	0.27	2.15	2.39
τ41	0.00	0.00	−0.05	0.05	0.05	0.00	0.10	0.06	0.04	0.08	0.23	0.17	0.29
τ42	1.00	0.00	0.94	1.06	0.06	1.00	1.12	0.04	1.02	1.05	0.26	1.19	1.34
τ43	2.00	0.00	1.92	2.09	0.04	1.96	2.13	−0.01	1.98	2.01	0.23	2.14	2.33
λ2	0.80	0.00	0.73	0.87	−0.02	0.71	0.85	−0.12	0.65	0.71	−0.07	0.66	0.79
λ3	1.00	0.00	0.91	1.09	−0.03	0.88	1.06	−0.15	0.81	0.89	−0.11	0.80	0.97
λ4	0.70	0.00	0.64	0.76	0.00	0.64	0.76	−0.06	0.61	0.67	−0.01	0.63	0.76
σ^2^(F)_US	1.60	0.01	1.39	1.84	0.44	1.77	2.32	0.69	2.16	2.44	2.31	3.34	4.53
σ^2^(v1)_HK	1.00	0.01	0.92	1.10	−0.05	0.87	1.04	−0.02	0.90	1.06	−0.19	0.73	0.90
σ^2^(v2)_HK	1.00	0.00	0.90	1.10	−0.12	0.79	0.96	−0.31	0.62	0.77	−0.38	0.56	0.69
σ^2^(v3)_HK	1.00	0.00	0.90	1.10	−0.10	0.80	0.99	−0.31	0.62	0.76	−0.36	0.57	0.72
σ^2^(v4)_HK	1.00	0.00	0.93	1.08	−0.09	0.84	0.98	−0.22	0.71	0.84	−0.30	0.65	0.76
μ(F)_HK	0.90	0.00	0.84	0.96	0.11	0.94	1.07	0.22	1.07	1.17	0.47	1.28	1.48
σ^2^(F)_HK	1.00	0.01	0.88	1.14	−0.06	0.82	1.07	−0.04	0.87	1.05	−0.23	0.67	0.90

*The parameters not labeled with US or HK are constrained equal across the two groups*.

**Table 6B:** **Summary of parameter estimates for models with strict invariance across eight different percentages of contaminated data (Conditions 5–8)**.

**Strict invariance**		**M10–20**			**M20–30**			**M30–30**			**M40–40**		
**Parameters**	**True**	**Bias**	**90% CI of estimates**		**Bias**	**90% CI of estimates**		**Bias**	**90% CI of estimates**		**Bias**	**90% CI of estimates**	
τ11	0.00	0.29	0.21	0.38	0.50	0.40	0.61	0.46	0.36	0.58	0.77	0.63	0.91
τ12	2.00	0.42	2.30	2.55	0.74	2.59	2.90	0.80	2.64	2.97	1.24	3.04	3.46
τ13	3.00	0.42	3.26	3.59	0.79	3.61	3.99	0.92	3.71	4.13	1.45	4.19	4.71
τ21	0.00	0.21	0.15	0.28	0.35	0.27	0.43	0.31	0.23	0.39	0.52	0.41	0.63
τ22	1.00	0.29	1.21	1.38	0.52	1.42	1.62	0.57	1.46	1.68	0.91	1.76	2.05
τ23	1.50	0.36	1.77	1.96	0.68	2.06	2.30	0.82	2.19	2.45	1.28	2.61	2.95
τ31	0.00	0.26	0.19	0.34	0.44	0.35	0.54	0.41	0.31	0.50	0.69	0.55	0.82
τ32	1.00	0.34	1.24	1.45	0.62	1.49	1.75	0.68	1.55	1.82	1.09	1.91	2.27
τ33	2.00	0.40	2.28	2.53	0.76	2.61	2.92	0.92	2.76	3.10	1.45	3.24	3.67
τ41	0.00	0.20	0.15	0.26	0.33	0.27	0.40	0.29	0.22	0.36	0.49	0.39	0.58
τ42	1.00	0.29	1.22	1.37	0.52	1.43	1.62	0.57	1.47	1.67	0.89	1.77	2.02
τ43	2.00	0.33	2.23	2.42	0.61	2.50	2.73	0.72	2.60	2.85	1.14	2.98	3.30
λ2	0.80	−0.03	0.71	0.84	−0.04	0.70	0.84	−0.03	0.70	0.84	−0.03	0.69	0.85
λ3	1.00	−0.05	0.87	1.03	−0.07	0.85	1.02	−0.06	0.85	1.03	−0.06	0.84	1.04
λ4	0.70	0.01	0.65	0.77	0.01	0.65	0.77	0.01	0.65	0.78	0.02	0.65	0.79
σ^2^(F)_US	1.60	2.21	3.36	4.32	4.26	5.13	6.70	4.62	5.40	7.12	8.16	8.37	11.26
μ(F)_HK	0.90	0.41	1.22	1.39	0.72	1.52	1.73	0.79	1.57	1.81	1.23	1.98	2.30
σ^2^(F)_HK	1.00	0.01	0.89	1.15	0.00	0.87	1.14	−0.03	0.84	1.11	−0.05	0.82	1.10

*The parameters not labeled with US or HK are constrained equal across the two groups*.

#### Thresholds τs

*Thresholds* τs are the next parameter to be constrained to achieve a higher level of MI, namely SI. The invariance of thresholds is the second necessary requirement for comparison of latent means (Muthén and Christoffersson, 1981; Millsap and Yun-Tein, 2004). Taking a closer look at τs from Tables 3–6, we found estimates of thresholds for M0 models using SI and STRI are almost perfect, more accurate than those obtained using CI and WI. This result is reasonable as thresholds are constrained equal across the two groups in SI and STRI models, which is consistent with the true population setting and with fewer parameters being estimated. Comparing threshold estimates for the two groups using CI and WI, estimates for the US group are closer to the true values, while threshold estimates for the HK group tend to be more biased. Inspecting τ estimates in each table, we generally find that, as the percentage of contaminated cases increases, most thresholds are more inclined to be over- or under-estimated. In other words, threshold estimates are more biased as more contaminated cases were included.

#### Residual variances

*Residual variances* were fixed to 1 in the US group for model identification purposes, whereas they were freely estimated for the HK group in models with CI, WI, or SI constraints. Estimates in SI and WI models are reasonable although they are apparently deflated in the WI models as the percentage of RS contamination goes up, and slightly inflated in SI models. Some extremely large estimates of residual variances were found in less than five simulatations under CI. However, the highly distorted estimates for residual variance were in models holding CI. As said earlier, those extreme cases may be caused by insufficient sample size or more likely because no reasonable starting values were used in estimating most parameters among the four types of MI models.

#### Factor means and factor variance

*Factor means and factor variance* difference between groups usually are researchers' focal interest when conducting cross-nation comparisons like the example data we used. Small percentages of contamination may not prevent models from achieving MI, and therefore researchers may compare two groups in terms of the two types of factor parameters. As shown in Tables 3–6, they could be estimated accurately if no contaminated cases were included. But as the percentages of NERS and ERS cases increase, they tend to be more biased.

## Discussion

The pervasiveness of survey research using Likert-type scales in a multitude of disciplines is generally unquestioned. But the various RSs possibly employed by participants taking these surveys and their potential effects on statistical modeling such as MI have long been ignored, unassessed or simply not reported. As many researchers have noted, the validity of MI from survey data is necessary to ensure that the results of cross-group comparison studies involving latent factors will be appropriate, meaningful, and interpretable.

We have observed that many studies examined MI directly but fail to consider or examine RSs. Only a few studies tried to address RSs together with MI. As they studied RSs using real data, given the fact that the researchers did not know the true nature underlying the data, applied researchers may assume RSs do not have substantial deleterious effects and consequently not apply any effort to detect and correct for these effects.

Our simulation results clearly demonstrated that the magnitude of these effects is not neglible, especially when the proportion of contaminated cases is not too small. Researchers should not ignore RSs such as ERS and NERS because they have significant impact on both model fit and parameter estimates. Five percent of cases contaminated by RSs may not have a significantly detrimental impact to the four levels of MI. This finding indicates model fit indices are not very sensitive to the inclusion of small proportion of RSs, exhibiting certain degree of resilience. Ten percent contamination may further cause STRI to fail, even though the true data held this property. Larger percentages of contamination may result in more severe distortion of model fit results, and in turn, rejection of theoretically correct MI models.

It appears that CFI and TLI are not helpful in detecting model misfit when RSs are introduced to data as they almost always suggest sufficient fit to data. RMSEA can detect the effect of RSs only when the percentages of RSs is very large, such as 40 percent in our case. The Chi-square test family, including Chi-square, Chi-square difference test, and relative Chi-square test, are much more sensitive to the inclusion of contaminated data when the percentages of contamination reach 10 or 20 percent. WRMR may be the most sensitive index because it can alert researchers to model misfit when the percentage reaches 20 for SI models or 10 or less for STRI models.

The effect of varying RSs on parameter estimation is even more substantial. Even five percentages of RS contamination can lead to biased estimates for the factor loadings, the thresholds, residual variance and factor means and variance. Generally, researchers would expect that the larger percentage of contaminated cases, the larger the estimation bias, as evidenced by the simulation results. The empirical 90% confidence intervals of parameter estimates also confirm that the bias of estimates are not ignorable. Moreover, the difference between parameter estimates of two groups was increased by RSs, which directly threaten the validity of MI and render meaningless group comparison by obscuring or exaggerating actual group differences.

This study is expected to have important implications for researchers or practitioners who are interested in studying construct comparability across groups. We would suggest that the examination of the possible presence of RSs should be routine when testing for MI. Although we have not finished examining all possible RSs, the evidence presented here is sufficient to alert researchers to the possible negative effects brought about by the presence of ERS and NERS or potentially other unexamined RSs. The finding of the current study is informative for practitioners to determine what percentages of NERS and ERS may present a serious threat to MI and which model fit indices are more sensitive to detect the RSs in the data. As our original model without RS contamination meets the highest level of MI, STRI, it is reasonable to predict that if the same percentage of contaminated cases were added to the data, to which SI or WI is assumed, the estimated model fit and parameter estimate results would be even worse than what were presented here.

## Author contributions

Estimate of contributions: ML: conceptualization, 60%; writing the article, 80%; analyzing data, 100%. AGH: conceptualization, 30%; writing the article 20%. JRH: conceptualization, 5%. GRH: conceptualization, 5%.

### Conflict of interest statement

The authors declare that the research was conducted in the absence of any commercial or financial relationships that could be construed as a potential conflict of interest.
